# Lipids, Gut Microbiota, and the Complex Relationship with Alzheimer’s Disease: A Narrative Review

**DOI:** 10.3390/nu15214661

**Published:** 2023-11-03

**Authors:** Daiane Oliveira Simão, Vitoria Silva Vieira, Jéssica Abdo Gonçalves Tosatti, Karina Braga Gomes

**Affiliations:** 1 Faculty of Medicine, Federal University of Minas Gerais, Professor Alfredo Balena Avenue, 190, Santa Efigênia, Belo Horizonte 30130-100, MG, Brazil; daianeor7@gmail.com; 2 Department of Nutrition, School of Nursing, Federal University of Minas Gerais, Professor Alfredo Balena Avenue, 190, Santa Efigênia, Belo Horizonte 30130-100, MG, Brazil; vitoriasilvavieira4067@gmail.com; 3 Department of Clinical and Toxicological Analyzes, Faculty of Pharmacy, Federal University of Minas Gerais, Presidente Antônio Carlos Avenue, 6627, Pampulha, Belo Horizonte 31270-901, MG, Brazil; jesstosatti@gmail.com

**Keywords:** lipids, gut microbiota, diet pattern, Alzheimer’s disease

## Abstract

Alzheimer’s Disease (AD) is a multifactorial, progressive, and chronic neurodegenerative disorder associated with the aging process. Memory deficits, cognitive impairment, and motor dysfunction are characteristics of AD. It is estimated that, by 2050, 131.5 million people will have AD. There is evidence that the gastrointestinal microbiome and diet may contribute to the development of AD or act preventively. Communication between the brain and the intestine occurs through immune cells in the mucosa and endocrine cells, or via the vagus nerve. Aging promotes intestinal dysbiosis, characterized by an increase in pro-inflammatory pathogenic bacteria and a reduction in anti-inflammatory response-mediating bacteria, thus contributing to neuroinflammation and neuronal damage, ultimately leading to cognitive decline. Therefore, the microbiota–gut–brain axis has a significant impact on neurodegenerative disorders. Lipids may play a preventive or contributory role in the development of AD. High consumption of saturated and trans fats can increase cortisol release and lead to other chronic diseases associated with AD. Conversely, low levels of omega-3 polyunsaturated fatty acids may be linked to neurodegenerative diseases. Unlike other studies, this review aims to describe, in an integrative way, the interaction between the gastrointestinal microbiome, lipids, and AD, providing valuable insights into how the relationship between these factors affects disease progression, contributing to prevention and treatment strategies.

## 1. Introduction

Alzheimer’s disease (AD) is a progressive, chronic, multifactorial, and irreversible neurodegenerative disorder associated with aging and functional decline [1,2]. It represents a global public health concern, being the most common cause of dementia in the elderly worldwide, affecting approximately 13% of individuals aged 75 to 84 and 33.3% of those aged 85 or older. Moreover, it ranks as the fifth leading cause of death among Americans aged 65 or older [3]. Projections indicate that, by 2050, an estimated 131.5 million people will be living with AD [2].

The disease manifests through the gradual deterioration of cognitive function, including memory loss, language, reasoning, and skills, as well as personality changes, impairing activities of daily living and making the individual increasingly dependent [3,4]. The progression of AD can be categorized into stages: (1) The first stage is the preclinical phase, which occurs before the onset of noticeable clinical symptoms. During this phase, the characteristic brain changes of the disease already occur, but there are no evident behavioral alterations yet. (2) The next stage is mild cognitive impairment, characterized by mild memory lapses, difficulty finding words, and forgetting recent events. However, daily activities remain unaffected. (3) In the most advanced stage, dementia proper occurs, where there is an inability to recognize close individuals, and cognitive and behavioral functionality is significantly impaired [4].

Current treatments have limited efficacy and often come with various adverse effects, typically providing short-term improvements in quality of life. Available medications are based on symptomatic therapy and the disease stage. Drugs such as antipsychotics, antidepressants, sedative and hypnotic agents, and mood stabilizers are used to manage the behavioral and psychological symptoms [4,5,6].

The pathology of AD is complex, but it is known to be related to the accumulation of extracellular amyloid β-peptide (Aβ) and intracellular neurofibrillary tangles (NFTs) with hyperphosphorylated tau protein [7]. Evidence suggests that Aβ contributes to neuronal damage, oxidative stress, reduced synaptic plasticity, and neuroinflammation [4,8]. The deposition of Aβ and NFTs leads to the loss of synapses and neurons, resulting in reduced responses to many neurotransmitters that control memory, reasoning, recognition of sensory stimuli, abstract thinking, and brain atrophy [9].

The triggering molecular mechanisms for increased Aβ production have been investigated. Genetic variants in the amyloid precursor protein (*APP*) genes on chromosome 21q21, presenilin 1 (*PSEN1*) on 14q24.3, and presenilin 2 (*PSEN2*) genes on 1q31-q42 are associated with early onset AD (before the age of 65). These genetic pathogenic variants contribute to the formation of amyloid accumulation in the brain [10]. Moreover, polymorphisms in apolipoprotein E (*APOE*) gene confer variable risks for AD, with the *APOE* ε4 allele being associated with late-onset AD (individuals aged 65 or older) [4,10].

Conditions such as dyslipidemia, hypertension, and type 2 diabetes mellitus can contribute to the development of AD, involving mechanisms such as hypoperfusion, insulin resistance, inflammation, hyperglycemia toxicity, and cerebrovascular damage [10,11]. In addition, stroke causes dysfunction of the blood–brain barrier, reduced cerebral blood flow, and the accumulation of neurotoxic substances in the brain. It increases the production and processing of APP, which, in turn, increases the production of the β-amyloid peptide, facilitating amyloid accumulation in the brain, amplifying neuronal dysfunction, and accelerating the process of neurodegeneration [10]. Interestingly, electronic devices are also potential risk factors for AD. Microwaves interact with the human body, promoting electron and ion vibration, which collide with other molecules within the organism. This results in the creation of an electromagnetic field that produces various effects in biological systems [10]. Consequently, microwaves can harm the brain, as exposure to their radiation leads to changes in synaptic structure, the release of amino acid-based neurotransmitters, and calcium influx [10,12]. In fact, experimental studies have shown a significant reduction in learning and memory capacity, similar to neuronal degeneration, and the enlargement of spaces around blood vessels in the hippocampus, causing adverse effects that may contribute to the development of AD. Other potential modifiable risk factors for AD are diet, physical inactivity, inadequate sleep, stress, inflammation, smoking, alcohol consumption, and an imbalance of the gastrointestinal microbiome (GM) [5,13,14].

Evidence supports that the GM plays a role in the pathogenesis and progression of AD, potentially acting preventively, mitigating the disease’s symptoms, or being one of the causal factors [15,16]. It is composed of a diverse community of microorganisms, including viruses, archaea, protozoa, fungi, and bacteria, which are capable of influencing brain functions through afferent signaling pathways and the secretion of biologically active substances [8,13]. This complex network of microorganisms plays a fundamental role in nutrient digestion and absorption, as well as in the development of the immune system. Bacteria perform vital functions in the fermentation of indigestible carbohydrates, vitamin production, and protection against pathogenic invaders [17].

Studies showed that the aging process is associated with an imbalance among GM microorganisms, known as intestinal dysbiosis, which is a risk factor for AD through the activation of neuroinflammation and amyloid formation [16,18]. Furthermore, several lifestyle factors play a crucial role in affecting the GM, influencing its composition and balance throughout life. Diet, in turn, has a direct link to intestinal modulation, and fat intake can act as a risk factor or prevent AD by modulating GM composition [19].

This narrative review aims to discuss the available scientific evidence on the interaction between GM, lipids, and AD, providing insights into how the interplay between these factors affects the disease’s progression, potentially aiding in prevention and treatment strategies. Given that a 10 to 25% reduction in risk factors can prevent up to 1.1 to 3.0 million cases of AD globally [20], knowledge of the controllable risk factors of AD can reduce morbidity and mortality from the disease worldwide.

## 2. Materials and Methods

A search was conducted in the following databases: Medical Literature Analysis and Retrieval System Online (MEDLINE) via PubMed, Cochrane Central Register of Controlled Trials (CENTRAL), Embase, Scopus, and Web of Science. The search utilized Medical Subject Headings (MeSH) terms and their corresponding entry terms in both Portuguese and English: “Lipids AND Gut Microbiota AND Alzheimer”. The inclusion criteria for article selection encompassed publications in Portuguese, English, or Spanish that addressed the relevant topic and were indexed in the databases. Studies that did not specifically pertain to AD were excluded.

## 3. Results

### 3.1. Intestinal–Brain–Microbiota Axis in Alzheimer’s Disease

The intestinal microbiota plays a fundamental role in overall host health, influencing various aspects of metabolism, the immune system, and behavior. Its impact on health is significant due to its integral role in the functioning of the human body. Currently, the GM has garnered interest from researchers in the field of health, especially in the context of neurodegenerative diseases [21]. Through its influence on the central nervous system (CNS), the term “gut–brain–microbiota axis” (GBMA) has been proposed [8,13].

Communication between the CNS and the gastrointestinal tract (GI) is constant and bidirectional, occurring through neural, immune, endocrine, and metabolic signaling, influencing both brain function and gut health. It can significantly contribute to the development and progression of AD under conditions of imbalance [8,22,23]. The CNS controls the permeability, secretion, movement, and immunity of the GI through efferent autonomic nerve pathways, conditioning the enteric nervous system and intestinal mucosa. Meanwhile, the GM can modulate brain functions through signaling pathways and the release of active substances [8].

The neural pathway occurs through the vagal circuit, which plays a fundamental role in connecting the GBMA through afferent and efferent pathways via the vagus nerve (cranial nerve). The afferent pathway is activated by the gut microbiota and intestinal metabolites, resulting in the generation of action potentials that lead to the release of the neurotransmitter glutamate, mainly excitatory, in the nucleus of the solitary tract, which has a neurotoxic effect in excess [24]. These signals are sent by the vagus nerve to the brain, conveying information about the condition of the gut, such as the presence of beneficial or pathogenic bacteria and the production of inflammatory substances. On the other hand, the efferent pathway releases acetylcholine, which modulates the anti-inflammatory pathway and promotes the maturation of group 3 innate lymphoid cells, which synthesize protectin conjugates in tissue regeneration, acting as an anti-inflammatory mediator. The brain can send signals via the vagus nerve to influence GM activity and regulate digestive functions. These signals can affect gastrointestinal motility, the production of digestive enzymes, intestinal barrier permeability, and other important functions related to the gastrointestinal tract [22,24].

Any disruption in the communication between the gut and the brain can affect the immune system’s ability to combat pathogens, prevent chronic inflammation, and maintain CNS health. This communication is particularly relevant, since both inflammation and the immune response play a pivotal role in the origin of AD. The communication of the GBMA through the immune system causes chronic low-grade inflammation, which can persist over time without causing severe immediate symptoms. Immune cells present in the gut can release pro-inflammatory cytokines, such as tumor necrosis factor (TNF), interferon-gamma (IFN-γ), and interleukin (IL)-6, which can cross the blood–brain barrier (BBB) and affect the brain. The hypothalamic–pituitary–adrenal axis also contributes to the communication of the GBMA by releasing glucocorticoids and producing inflammatory cytokines in periods of stress, being the main endocrine signaling pathway [15]. Furthermore, the presence of metabolites produced by the intestinal microbiota, such as tryptophan and neurotransmitters, would be important for intestinal immune regulation. Tryptophan and its metabolites contribute to sleep regulation as precursors to melatonin and have anti-inflammatory properties, maintaining the intestinal barrier integrity and modulating the immune system [15].

Studies show that a healthy GM can modify behavior through the GBMA. Escherichia, Lactobacillus, Saccharomyces, and Bacillus can synthesize monoamines, short-chain fatty acids (SCFAs), gamma-aminobutyric acid (GABA), serotonin, and dopamine, which are metabolites that enhance cognition and memory [17]. SCFAs are produced by the fermentation of non-digestible carbohydrates, mainly by gram-positive bacteria, with acetate, propionate, and butyrate, which serve as protective factors for the GBMA. They provide energy to intestinal cells, promote intestinal barrier integrity, and prevent the proliferation of pathogenic bacteria associated with AD development by altering the intestinal pH, as well as stimulating anti-inflammatory mediators [25]. Furthermore, they can traverse the intestinal tract and the circulatory system, cross the BBB, and reach the brain, where they act as agonists of the aryl hydrocarbon receptor (AhR), a ligand-activated transcription factor in microglia and astrocyte cells, reducing inflammation, neurotoxicity, and immune cell chemotaxis [22].

The aging process itself leads to GM imbalance (intestinal dysbiosis), which negatively influences the brain through the production of pro-inflammatory cytokines and bacterial metabolites, contributing to the development and progression of age-related neurodegenerative disorders, such as AD [18,26]. In contrast to SCFA, intestinal dysbiosis in the elderly leads to reduced levels of butyrate [17] and weakened intestinal epithelium, causing the dissemination of intestinal microorganisms, brain inflammation, and cognitive impairment [27]. Increased inflammation by glial cells allows the accumulation of the Aβ peptide in the brain, one of the known pathophysiological factors of AD [9]. Thus, understanding the GBMA is essential for developing new therapeutic strategies for the disease.

### 3.2. Dysbiosis as a Risk Factor for Alzheimer’s Disease

The gastrointestinal tract (GI) has the largest reservoir of microorganisms in the human body. The intestine is the primary site for their presence, playing a crucial role in physiology. The term “symbiosis” is used to describe the delicate balance between beneficial and pathogenic microorganisms, which is essential for maintaining cerebral homeostasis [28]. According to Eckburg et al. (2005), the human intestinal microbiota is predominantly composed of the phyla Firmicutes and Bacteroidetes [29]. Intestinal dysbiosis, on the other hand, is characterized by a reduction in beneficial microorganisms and an increase in pathogenic ones, leading to symptoms such as abdominal distension with excessive gas production, constipation or diarrhea, digestive problems, and intestinal inflammation [30].

The composition of the gut microbiota varies from person to person and it is established during the early years of life, affecting the microbiota composition in adulthood. It is influenced by factors such as the mother’s lifestyle, type of childbirth, breastfeeding method, introduction of solid foods, and antibiotic use in infants [17,31]. During adulthood, factors such as diet, environment, diseases, antibiotic use, sedentary lifestyle, stress, and inadequate sleep also influence its composition, although it generally remains relatively stable. However, in the elderly, the balance and diversity of microorganisms tend to deteriorate, increasing the risk of age-related diseases [28,32,33].

Modifiable risk factors for AD also interact with the GM and contribute to the multifactorial nature of the disease. A sedentary lifestyle increases the risk of chronic diseases [34,35], such as obesity, diabetes, and cardiovascular diseases, compromising GM diversity and reducing competitive interactions among microorganisms [36], leading to intestinal dysbiosis. On the other hand, regular physical exercise provides protection for health, improving functional capacity and cognition [37,38,39]. An active lifestyle has the ability to modulate the GM, increasing its diversity and promoting a more robust and resilient GM. Additionally, it enhances intestinal transit, releases myokines, hormones, and neurotransmitters [36,40,41]. Conversely, the GM also improves exercise performance, energy metabolism, and colon health, and enhances the immune response [34,36]. Therefore, regular physical exercise in the elderly is protective against AD [40] and slows down the progression of the disease [38].

Stress is capable of disrupting body homeostasis [42] through the release of the hormone cortisol, and it has direct impacts on the GM [43,44] due to the dysregulation of the hypothalamic–pituitary–adrenal axis [44]. It is associated with intestinal dysbiosis [44,45], characterized by decreases in Bacteroides and Lactobacillus and an increase in the Clostridium genus [44], increased production of inflammatory cytokines [46,47], dysfunction of the intestinal barrier [48], and increased intestinal barrier permeability [49,50]. This disrupts the homeostasis of the GBMA axis, impairing cognition [51]. Furthermore, stress-related symptoms include mood disorders, fatigue, decreased performance, and insomnia [44], reducing the quality of life and promoting AD risk.

Smoking increases the risk of heart and respiratory complications, including atherosclerosis, coronary artery disease, chronic obstructive pulmonary disease, and emphysema [52]. Smoking is a risk factor for AD and affects the oral, respiratory, and gastrointestinal microbiotas [52]. Regarding the GM, smoking alters mucin production [53], increases the production of reactive oxygen species, causes dysbiosis [52], and increases intestinal barrier permeability [54]. Quitting smoking promotes intestinal health by increasing microbial diversity and reducing the presence of Proteobacteria and Bacteroidetes, as indicated by the longitudinal studies conducted by Biedermann et al. (2013) and Biedermann et al. (2014) over a cessation period of 4 to 8 weeks [55,56]. These changes lead to the gut microbiota resembling that of individuals who have never smoked.

In aging, it is common changes in eating habits that occur due to alterations in taste, a reduction in digestive enzymes, and peristaltic disorders that lead to a reduction in the variety of foods consumed and lower fiber intake [2,57]. Additionally, reduced physical activity, the use of multiple medications, including antibiotics, a weakened immune system, pre-existing health conditions, and reduced gastric acid production, which alters the pH and promotes bacterial overgrowth, are factors that negatively affect and contribute to the development of intestinal dysbiosis in the elderly [58,59]. Furthermore, the diversity of the elderly’s intestinal microbiota is influenced by their social contact [59].

Recent studies have highlighted differences in the gut microbiota (GM) composition between AD patients and healthy individuals [30,60]. Kaiyrlykyzy et al. (2022) investigated the diversity and composition of the GM in 41 elderly AD patients and 43 healthy elderly individuals in Kazakhstan, and they identified significant differences between the two groups at the phylum, class, order, and genus levels of microbiota. AD patients showed reductions in Bifidobacterium, Clostridia, Castellaniella, Erysipelotrichaceae, Roseburia, Tuzzerella, Lactobacillaceae, and Monoglobus [61]. Similarly, Zhuang et al. (2018) observed differences in Bacteroides, Actinobacteria, Ruminococcus, Lachnospiraceae, and Selenomonadales in the GM of AD patients compared with controls, based on fecal sample analysis [62]. A recent meta-analysis of eleven observational studies and pre-interventional data found that individuals with AD exhibited lower GM diversity compared with the control group. Additionally, they showed a higher abundance of Proteobacteria (which includes a wide range of pathogenic genera), Bifidobacterium, and Phascolarctobacterium, along with reductions in Firmicutes (bacteria involved in fiber fermentation and SCFA production), Clostridiaceae, Lachnospiraceae, and Rikenellaceae [63]. In the study by Vogt et al. (2017), the difference in GM composition between individuals with AD and controls was also elucidated. Analysis of fecal samples from participants in the Wisconsin Alzheimer’s Disease Research Center and the Wisconsin Registry for Alzheimer’s Prevention identified that AD patients showed a reduction in Firmicutes and Bifidobacterium and an increase in Bacteroidetes [64].

In addition to the changes associated with senescence, other factors, such as the aging of intestinal cells, a decrease in the mucosal layer, and an increase in spacing between intestinal cells contribute to dysbiosis in the elderly. This dysbiosis is characterized by a reduction in diversity and an increase in more pathogenic bacteria (Aleman and Valenzano, 2019). These alterations in the intestinal barrier promote the translocation of bacteria and their metabolites into the bloodstream, ultimately crossing the blood–brain barrier (BBB) and leading to the neuroinflammation observed in AD [65].

### 3.3. Intestinal Dysbiosis Disrupts the Homeostasis of the Gut–Brain–Microbiota Axis and Influences the Progression of Alzheimer’s Disease

To understand how intestinal dysbiosis disrupts the GBMA axis, it is necessary to grasp two concepts: the intestinal barrier and the blood–brain barrier (BBB). The intestinal barrier provides protection by preventing contact between the external environment and the internal milieu [66]. It is composed of the lamina propria (responsible for blood supply, lymphatic drainage, and nerve supply), enterocytes (intestinal epithelial cells held together by tight junctions), Paneth cells (responsible for producing antimicrobial peptides secreted into the mucosal layer), mucus (preventing bacterial adhesion), enteroendocrine cells (serotonin producers), and other immune system cells [66,67]. In homeostasis, commensal bacteria inhibit pathogen colonization [66] and the intestinal barrier is permeable to water and small molecules resulting from the digestion process [67]. Defects in intestinal barrier integrity will stimulate the immune system, cause inflammation, and make it more permeable by increasing the spacing between enterocytes, allowing the passage of larger molecules, such as bacteria and metabolites, reaching different organs, including the brain [67,68].

Similar to the intestinal barrier, the BBB also protects the brain, separating the CNS from the peripheral circulation. Composed of brain cells interconnected by tight junctions that strictly control the movement of molecules and nutrients between the blood and the brain, it allows the entry of essential brain nutrients (nutrient ions and oxygen) and the removal of potentially harmful substances for the proper functioning of neurons [69]. Compromising this barrier will uncontrollably increase the entry of neurotoxic substances, immune system cells, ions, and, in intestinal dysbiosis, bacteria and their metabolites, affecting brain health. Intestinal dysbiosis contributes to the progression of AD pathology through mechanisms that stimulate the formation of amyloid plaques and neuroinflammation [21].

The production of amyloid proteins by bacteria associated with intestinal dysbiosis serves as protection for these bacteria through the formation of biofilms. These bacterial amyloids share structural similarities with those found in the brains of individuals with AD. They can trigger inflammatory responses in the intestine and the body, affecting the immune system and even the CNS, which are factors associated with the development and progression of AD [23]. Deposits of Aβ were identified in post mortem examinations of the intestines of AD patients [70]. Although the mechanisms involved in this condition are not yet fully clear, dysbiosis may contribute to the accumulation of APP in the intestine, even in the early stages of the disease [71]. Furthermore, bacterial amyloid proteins may potentially influence the process of amyloid protein aggregation in the brain, which is also a hallmark of the disease. Exposure to bacterial amyloid proteins in the gut can lead to the so-called “priming” of the immune system, resulting in a more intense reaction to the endogenous production of amyloid proteins in the brain [23].

Intestinal dysbiosis can also amplify the generation of substances like trimethylamine N-oxide (TMAO), which, by increasing the activity of the β-secretase enzyme, contributes to the exacerbation of Aβ accumulation in the brain, furthering the progression of AD [72]. The elevation of bacterial bile acids can breach the BBB, allowing the entry of peripheral cholesterol into the CNS, also stimulating the formation of Aβ and AD progression [73,74]. Dysbiosis can lead to the hyperstimulation of the immune system, resulting in the production of inflammatory cytokines. In older individuals, this dysregulated immune response can cause low-grade inflammation and atrophy of intestinal mucosal cells, and facilitate bacterial translocation and cytokine leakage into the bloodstream. These substances and bacteria can breach the BBB, intensifying neuroinflammation by activating receptors on neurons, glial cells, microglia, and macrophages in the brain. This process constitutes a crucial aspect of AD pathogenesis, promoting and exacerbating the disease [13,23,28,30,75].

It has been elucidated that AD patients have a higher population of gram-negative bacteria. Gurav An. (2019) confirmed the presence of gram-negative bacteria in the peripheral nervous system and the CNS of AD patients through 16S ribosomal DNA sequencing in autopsy brain samples from individuals with AD compared with those without AD, matched by age [76]. This suggests that these bacteria can infiltrate the brain through the BBB. Moreover, Beydoun et al. (2020) demonstrated that the presence of these bacteria was associated with higher mortality related to AD in a retrospective cohort study [77].

Gram-negative bacteria secrete lipopolysaccharide (LPS), an endotoxin composed of a lipid and a polysaccharide found in the outer membrane of bacteria, which provides structural protection [15]. Released LPSs can disrupt the integrity of the intestinal barrier, making it permeable and inducing neuroinflammation and microglial activation, which trigger various pathways in AD, including beta-secretase 1 [78]. LPS binds to TLR4 receptors, leading to the activation of the NF-κB pathway, which, in turn, induces the expression of genes associated with pro-inflammatory cytokines [79].

Studies have shown higher levels of LPS in amyloid plaques in the superior lobe and hippocampus of AD patients compared with control individuals [4,80]. While the complete mechanism by which LPS crosses the BBB remains unclear, Kim et al. (2021) proposed four possible mechanisms to explain this phenomenon. One hypothesis suggests that LPS aggregates with binding proteins that utilize their own BBB receptors to facilitate crossing. Another mechanism involves LPS being transported through phagocytosis from peripheral immune cells. This occurs because LPS tends to increase the production of inflammatory cytokines and adhesion molecules in BBB cells, making it easier for immune cells to enter the CNS [81]. Additionally, there is the idea that an excess of LPS could compromise the BBB’s integrity, causing damage through induced inflammatory cytokines. Such damage would enable LPS to passively enter the CNS [80]. Furthermore, LPS might directly enter the CNS by binding between CD-14 and TLR4 receptors, potentially resulting in neurological damage. Lastly, the entry of LPS into the CNS via transporters associated with gram-negative bacteria, which carry LPS along, could potentially lead to neuronal damage and eventual neural death. While these mechanisms have been proposed as potential ways by which LPSs cross the BBB, it is important to note that there are still gaps in our understanding, and further research is needed to fully elucidate this complex process [81].

LPS is directly linked to neurodegeneration in AD as it triggers neuronal loss and affects synaptic and cognitive function. This relationship arises from the damage caused to myelin and its impact on CNS synapses. Excess LPS contributes to the establishment of a state of neuroinflammation. Moreover, LPS is also implicated in promoting the hyperphosphorylation and aggregation of tau protein in the brain, intensifying the neurodegenerative deterioration seen in AD [81]. Thus, LPS can influence both the onset and progression of the disease. Consequently, maintaining symbiosis within the body is essential for slowing down the progression of AD, potentially acting preventively in the development of the disease [28]. Therefore, modulating the intestinal microbiota is an important therapy, with diet being one of the primary factors influencing the intestinal microbiota, which will be discussed next.

### 3.4. Brain Composition and Lipids

Lipids are macromolecules present in the human body, including the brain, and they serve crucial functions in cell membranes and signal transduction. These molecules are found in both animal and plant-based foods. Remarkably, the human brain contains a substantial amount of lipids, which play vital roles in brain structure and function. In fact, it ranks as the second organ with the highest lipid content, comprising at least half of its dry weight [82]. Brain lipids consist of approximately 50% phospholipids, less than 40% glycolipids, around 10% cholesterol, along with cholesterol esters, and trace of triglycerides and long-chain fatty acids [82]. Changes in lipid metabolism are believed to contribute to AD, as lipid oxidation in the brain has been identified as an early event in the disease [83].

Another critical component of cell membranes is cholesterol, which contributes to membrane fluidity. When neurons lack an adequate supply of cholesterol, it can compromise synaptic plasticity and nerve signal transmission, while also triggering tau pathology and neurodegeneration. Furthermore, the aging process and reductions in cholesterol levels have been linked to deteriorations in signal transmission, synaptic loss, increased tau pathology, and cell death [82]. Roy et al. (2004) conducted an analysis using brain tissue samples from seven AD patients and seven controls aged 68 to 93. They found elevated levels of free cholesterol and reduced sphingomyelin in the middle frontal gyrus of AD patients. These changes were associated with membrane oxidative stress and, consequently, AD [83].

The absence of a sufficient supply of cholesterol to neurons can compromise synaptic plasticity and nerve signal transmission, leading to tau pathology and neurodegeneration. Conversely, an increase in cholesterol levels has been linked to the generation of Aβ and has been detected in the early stages of AD patients. Therefore, contradictory data regarding the cholesterol concentration and its impact on AD may be explained by differences in disease stages [82].

It is essential to emphasize that, in addition to cholesterol and phospholipids, gangliosides are also present in the plasma membranes of nervous tissue, where they play a significant role in memory formation and nerve signal transmission [84]. In a study involving five AD patients and three controls, a significant reduction in all analyzed gangliosides was observed in the basal telencephalon, as well as in the frontal and temporal cortexes of individuals with AD. These findings suggest possible degeneration of cortical neurons, leading to the loss of memory, language, and abstract thinking functions [82].

It is well-established that AD becomes more prevalent as individuals age, and during this stage of life, changes in lipid composition may contribute to the disease [82]. In a study that exclusively included male individuals, in order to eliminate the potential confounding factor of female hormones, the authors examined 26 healthy young individuals under 30 years of age and 21 healthy middle-aged individuals over 50 years of age. Using gas chromatography, they identified variations in the plasma concentrations of different fatty acids. It became evident that older men had higher levels of total cholesterol and LDL-c, whereas younger men had higher concentrations of HDL-c and triglycerides [85]. Older men also exhibited significant differences in the total amounts of saturated, monounsaturated, and polyunsaturated fatty acids in their plasma. Notably, there was a significant increase in concentrations in middle-aged adults of saturated fatty acids, such as C14:0 and C24:0, monounsaturated fatty acids, such as C14:1, C18:1, and C24:1, and polyunsaturated fatty acids, like C18:3n6, C22:3n6, and C22:2. Concurrently, circulating concentrations of pro-inflammatory cytokines TNF and IL-6 increased with age, while concentrations of anti-inflammatory cytokines IL-10 and transforming growth factor beta 1 (TGF-β1) decreased. Healthy individuals aged 50 years displayed an increase in plasma free fatty acids and a plasma lipid profile characterized by medium-chain fatty acids [85].

Changes in blood lipid levels can indirectly influence the lipid composition in the brain, reflecting alterations in metabolic and inflammatory processes that also affect the brain. Specific blood lipids can impact the permeability of the blood–brain barrier, potentially influencing the availability of fatty acids and other lipids to brain cells by modifying the composition of the neural membrane [86]. Lipid microdomains are membrane structures composed of sphingolipids, cholesterol, and saturated and polyunsaturated fatty acids. These microdomains play crucial roles in AD pathology, including the production of Aβ, which is closely related to the lipid composition of these structures. Additionally, lipid microdomains facilitate interactions among Aβ, APOE, and tau proteins, contributing to the aggregation of Aβ and the hyperphosphorylation of tau [82]. Further research is warranted to fully understand the significance of the brain lipid composition in preventing and treating AD.

### 3.5. Dietary Lipids and Alzheimer’s Disease

Fatty acids are classified based on the number of double bonds and can be categorized as saturated or unsaturated. Saturated fatty acids, which have no double bonds, tend to be solid at room temperature and are commonly found in animal products, like meats and dairy. Unsaturated fatty acids, on the other hand, contain at least one double bond and are further classified as monounsaturated (MUFA) or polyunsaturated (PUFA) when they have two or more double bonds. Fruits and vegetables are primary sources of unsaturated fats [82].

The fast-paced lifestyle of modern society has given rise to various health-damaging factors. In this context, the Western diet plays a significant role. This diet is characterized by high consumption of fast food, processed meats, refined grains, and frozen foods, resulting in increased intake of calories, sugars, additives, saturated fats, and trans fats, while often lacking in fruits and vegetables. The prevalence of ultra-processed foods, which are cost-effective and heavily marketed, further promotes this dietary pattern [87]. The adverse effects of the Western diet are linked to elevated total tau levels and a profile consistent with cerebrospinal fluid biomarkers in the preclinical phase of AD [88]. The Western dietary pattern, associated with altered nutritional status (excess weight), may contribute to a chronic low-grade inflammatory response, as observed in AD [25].

High consumption of saturated fats is associated with a higher risk factor for chronic and low-grade systemic inflammation. This is due to their ability to increase the concentration of gram-negative bacteria in the gut, subsequently elevating the levels of LPS in the body. As previously described, LPS is involved in initiating and progressing inflammation through its impact on intestinal permeability [89]. Moreover, the metabolism of saturated fatty acids can generate reactive oxygen species, causing harm to the intestine and exacerbating neurodegeneration in individuals with AD [19,89]. Gubert and colleagues (2020) demonstrated that increased saturated fat consumption induced an inflammatory response by peripheral immune cells that could be transported to the CNS. Furthermore, an increased intake of fats, particularly saturated fats, combined with other risk factors, such as a sedentary lifestyle and alcohol consumption, was associated with potential adverse health effects. Depending on the nature and quantity of lipids in the diet, this may be linked to low-grade widespread inflammation mediated by inflammatory markers in adipose tissue, such as TNF and IL-6. Moreover, this exacerbated inflammation, as already observed in AD, could potentially contribute to the disease’s progression [19].

As dietary habits change, studies have become increasingly interested in understanding their relationship with AD pathogenesis. The brain’s lipid complexity plays fundamental roles in neural development, differentiation, nerve cell migration, and synaptic processes, as previously described. Furthermore, lipids are integral to the composition of neural membranes, crucial for the proper functioning of the nervous system [86].

Trans fatty acids are formed through the hydrogenation of various fatty acids found in vegetable oils. They are commonly used by the food industry to extend the shelf life of products, improve their texture, and flavor [45]. Trans fats exhibit hypercholesterolemic effects and are strongly associated with an increased risk of cardiovascular diseases, consequently promoting a greater risk of vascular dementia. Both saturated and trans fats are often referred to as “bad fats” due to their detrimental health effects. An elevated risk of developing AD and dementia was observed in elderly individuals with high consumption of trans fatty acids, particularly in the context of a Western lifestyle [88]. Morris et al. (2003) conducted a longitudinal community-based study involving participants from the Chicago Health and Aging Project, with a focus on identifying risk factors for AD development in individuals aged sixty-five or older. Dietary intake was assessed through a self-administered food frequency questionnaire that included more than 139 foods and the use of vitamin supplements. Their findings revealed a positive association between trans fatty acid intake and the risk of AD. Over a mean follow-up period of 3.9 years, 131 individuals developed AD [90]. Moreover, a recent meta-analysis by Ruan et al. (2018) that included four independent prospective cohort studies emphasized that a high consumption of saturated fats is linked to a 39% increase in AD risk, with an even higher risk (105%) associated with the development of any type of dementia [91].

Omega-3 fatty acids include alpha-linolenic acid (ALA), eicosapentaenoic acid (EPA), and docosahexaenoic acid (DHA). ALA is considered an essential fatty acid, which means that the human body cannot produce it in sufficient quantities, necessitating its acquisition through diet. EPA and DHA can be converted from ALA through chain elongation and desaturation [92]. ALA is found in foods such as flaxseeds, chia seeds, and walnuts, while EPA and DHA, the primary bioactive forms in humans, are present in fish, fish oil, and seafood [88]. EPA primarily plays a role in cell signaling, while DHA is crucial for cognitive functions, including learning and memory, due to its anti-apoptotic and anti-nociceptive effects, which impact nigrostriatal activities and synaptic plasticity [86,93]. DHA can reduce neuronal loss and improve learning and memory, countering brain atrophy and cognitive decline [93].

Omega-6 fatty acids have a double bond at the sixth carbon from the methyl end. The primary fatty acid in the omega-6 family is linoleic acid (LA), also an essential fatty acid that can be metabolized into arachidonic acid (AA) and gamma-linolenic acid (GLA). GLA is found in human milk and in borage oil, blackcurrant, and evening primrose oil, while AA is obtained from a diet rich in offal, poultry, and eggs [94].

The typical Western diet has a detrimental effect on the balance between omega-6 and omega-3 fatty acids, shifting it from an ideal 1:1 ratio to a less favorable 20:1 ratio [86]. This imbalance is primarily attributed to increased consumption of omega-6-rich vegetable oils, like corn oil, soybean oil, and sunflower oil, commonly used in the production of processed foods. This skewed omega-6/omega-3 ratio has been associated with various health issues, including obesity and type 2 diabetes mellitus, both risk factors for AD [92].

Omega-3 fatty acids in the diet exhibit anti-inflammatory properties by suppressing genes associated with inflammation and modifying cell membrane structure, displacing omega-6 polyunsaturated fatty acids and cholesterol, leading to changes in lipid raft organization [86]. They also help to reduce fat accumulation in adipose tissues by inhibiting lipid synthesis enzymes and stimulating β-oxidation [92]. The Mediterranean diet (MD), which includes fish as a significant component, provides adequate intake of essential fatty acids, like EPA and DHA, which interact with omega-3 and omega-6 metabolites in the brain, making the consumption of these fatty acids a promising option for potential AD treatment [86].

MUFAs, such as oleic acid found in olive oil, avocados, and nuts, have been linked to brain health benefits and a potential reduction in the risk of AD. They possess antioxidant and anti-inflammatory properties that contribute to overall health [53]. Extra-virgin olive oil, a rich source of oleic acid, has been studied for its potential role in preventing and slowing down the progression of AD. It accomplishes this by reducing Aβ deposits, mitigating tau neuropathology, enhancing Aβ clearance mechanisms through the blood–brain barrier (BBB), and decreasing astrocyte and microglial cell activation, resulting in reduced production of inflammatory cytokines. Additionally, the broad antioxidant benefits of extra-virgin olive oil can lead to increased brain antioxidant enzymes and reduced levels of reactive oxygen species [95].

Several studies have explored the potential benefits of omega-3 supplementation for mild cognitive impairment, but their findings have been somewhat contradictory due to variations in supplementation dosage and treatment duration [88,96,97]. For instance, a study by Lin et al. (2022) [96] found no statistically significant differences between the placebo and treatment groups in terms of cognitive function, mood state scores, biochemical profiles, and levels of inflammatory cytokines in their multicenter, randomized, double-blind, placebo-controlled study. Conversely, a systematic review of randomized clinical trials, conducted by Moral and Fortique (2019) [97], evaluated the relationship between omega-3 supplementation and cognitive status in older adults and younger adults. Ten out of fourteen analyzed studies showed positive results in at least one aspect of cognitive function, suggesting that omega-3 supplementation might have a favorable effect on cognitive function. This indicates that long-chain polyunsaturated fatty acids, such as omega-3s, could be considered as a preventive or therapeutic approach for addressing cognitive decline in older adults or the elderly. The study conducted by Stavrinou et al. (2020) [98], which involved supplementing older adults with mild cognitive impairment with a combination of omega-3 and omega-6 fatty acids, as well as vitamins A and E, showed promising results in cognitive function and functional capacity improvements. This significant finding supports the potential benefits of a balanced intake of these essential fatty acids and vitamins in promoting cognitive health [98].

In summary, the critical role of dietary fat composition in neuroinflammation is evident. Diets rich in omega-6 fatty acids, saturated fats, and trans fatty acids, such as the Western diet, tend to promote neuroinflammation. Conversely, diets that are abundant in monounsaturated fatty acids (MUFAs), omega-3 fatty acids, and sphingolipids have the potential to reduce neuroinflammation. Maintaining an appropriate balance between omega-3 and omega-6 fatty acids in the diet is crucial, as an excessive imbalance in favor of omega-6 can promote inflammation, while omega-3 fatty acids can help mitigate these effects [86]. This comprehensive explanation effectively summarizes the relationship between dietary fats, inflammation, and cognitive health, highlighting the importance of dietary choices in promoting brain health.

### 3.6. Impact of Dietary Lipids on Gut Microbiota

Dietary lipids play a significant role in influencing the positive or negative impact on the composition and diversity of the intestinal microbiota [88]. Sphingolipids sources are found in wheat, soy, eggs, and dairy. They are beneficial for intestinal health, as they can reduce inflammation and maintain crucial morphological characteristics for immunological homeostasis and intestinal functionality. In the GI, they compete for binding sites with commensal bacteria and provide a defense against pathogens [86].

On the other hand, high-fat diets with elevated levels of saturated, trans, and omega-6 fats have the ability to negatively influence the GI by promoting dysbiosis, altering intestinal permeability, changing the distribution and expression of tight junctions in the intestine due to the formation of reactive oxygen species as a product of their metabolism [19,86], and increasing the population of gram-negative bacteria that produce LPS [89]. Additionally, these fats have the potential to mimic the actions of LPS, which can influence intestinal health by triggering pro-inflammatory processes that result in the thinning of the mucus layer [86].

Chronic low-grade inflammation has been strongly implicated in the pathogenesis of AD, also associated with the activation of microglial macrophages in the brains of individuals with AD. This mechanism will result in the sustained production of pro-inflammatory cytokines, including IL-1β, IL-6, and TNF, perpetuating a cycle of neuro-inflammatory processes, including reduced brain volume, events related to cerebral vascular diseases, such as microbleeds and infarctions, and neuronal death [99]. On the other hand, the role of diet in modulating the immune system is well known in the literature, such as the intake of antioxidant vitamins and polyunsaturated fats that are capable of inhibiting oxidative stress and neuroinflammation, or the role of saturated and trans fatty acids in mediating pro-inflammatory processes, particularly in the hypothalamus [100].

In addition to the pro-inflammatory profile, a diet rich in saturated and trans fatty acids would be associated with the direct risk of AD [101] and the development of hypercholesterolemia [99], contributing to the accumulation of oxysterols in the brain in patients with AD [102]. It is still unclear whether the effects mediated by dietary profile on neurocognition are mediated by immunological mechanisms or by neuroinflammatory processes. However, a robust body of evidence suggests that changes in the gut microbiota as well as peripheral inflammation can amplify neuroinflammation and accelerate neurodegeneration, both mediated by a dietary profile rich in saturated and trans fatty acids [99].

Unlike saturated fat, omega-3 can directly modulate the diversity of the intestinal microbiota by increasing beneficial species that produce SCFAs and plays a role in the production of anti-inflammatory mediators [88]. Experiments conducted in animals have shown that the inclusion of fish oil in the diet has the effect of mitigating the deterioration of intestinal epithelial barrier integrity caused by the consumption of a high-saturated fat diet. These results indicate that omega-3 can reverse adverse changes in intestinal barrier function resulting from the consumption of a high-fat diet, as demonstrated in rodent experiments [86].

The omega-3 polyunsaturated fatty acids (ω-3) include ALA, EPA, and DHA. ALA is considered an essential fatty acid because it is not synthesized by humans, and EPA and DHA can be converted from ALA by chain elongation and desaturation. It is known that these PUFAs play a fundamental role in the production and storage of energy, synthesis and fluidity of cell membranes, and enzymatic activities [103].

In AD, PUFAs are necessary for the development, integrity, and function of the brain. In addition, they are important components of biomembranes, playing a fundamental role in the integrity, development, maintenance, and functions of neurons, including synaptic processes, differentiation, and neuronal growth [104]. Therefore, supplementation with ω-3 in the early stages of AD has a promising role in controlling its progression. In a review published by Bazan and collaborators, the positive relationship between a diet rich in ω-3, as in MD, and a better cognitive performance during aging was demonstrated [105].

### 3.7. Diet as a Modulator of Gastrointestinal Microbiome

Diet is recognized as a modifiable risk factor for AD, and dietary interventions have been studied as a therapy. The MD has gained popularity and is widely recommended as a globally healthy dietary pattern, proposed as an efficient way to prevent or delay the progression of AD. It is characterized by a varied diet based on fruits, vegetables, legumes, nuts, moderate consumption of fish and poultry, low to moderate consumption of red wine during meals, low consumption of dairy and red meat, and olive oil as the main source of fat [79,88,89]. This diet is also rich in fibers that are fermented by intestinal bacteria, resulting in the production of SCFAs with beneficial properties for intestinal and metabolic health. Furthermore, it is known for its anti-inflammatory properties as a source of MUFAs and PUFAs [86].

Another diet gaining visibility in the therapy for various diseases, including improving cognitive health and possibly preventing the development of dementia, like AD, is the plant-based diet. Similar to the MD, it primarily consists of foods from plant sources, including fruits, vegetables, grains, nuts, and seeds, with limitations on the consumption of animal products, such as meat, dairy, and eggs [106]. This dietary pattern contains essential functional nutrients with antioxidant, anti-inflammatory, and free-radical-fighting characteristics generated by oxidative stress that contribute to intestinal and brain health [107]. In a study that aimed to assess whether the MD could alter the intestinal microbiota, it was observed that the MD altered the intestinal microbiota with a higher Firmicutes/Bacteroidetes ratio and lower inflammatory markers [108]. It is estimated that most polyphenols from the MD diet are not absorbed in the small intestine, and their metabolites generated by microbes can inhibit the production and interfere with the misfolding of beta-amyloid peptides, as well as prevent the inflammation observed in AD [25]. However, when addressing a dietary pattern, it is important to consider not just a single nutrient, but also a variety of them. In this context, a diet centered on higher consumption of vegetables, whole grains, and fish has the potential to prevent intestinal inflammatory processes. It benefits from the synergistic action of nutrients with anti-inflammatory effects, reinforced by the intestinal microbiome that promotes the production of SCFA. Therefore, long-term diets tend to be more effective in mitigating the inflammatory effects resulting from changes in the intestinal microbiota [109].

**Figure 1:**
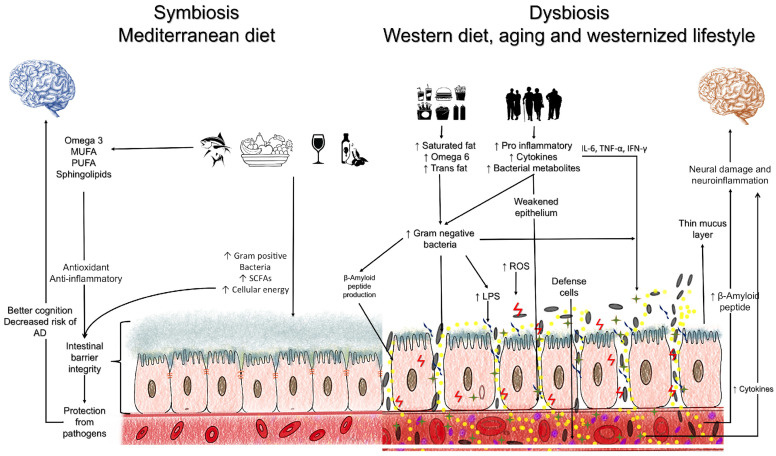
Main mechanisms involved in the relationship between the intestinal microbiota and Alzheimer’s disease.

Artificial sweeteners, emulsifiers, and food colorings constitute a class of food additives found in many ultra-processed foods. Discussing this point is important due to the high consumption of these compounds through the Western diet. Exposure to food additives can induce dysbiosis and dysregulation of intestinal homeostasis with alterations in the intestinal barrier [110]. These are risk factors for the development and progression of AD by promoting neuroinflammation and neurodegeneration, as previously elucidated. Furthermore, this reinforces the MD and plant-based diets as the ideal dietary patterns for the clinical therapy of AD (Figure 1). However, clinical trials involving these diets should be conducted to assess their effect on delaying the progression of AD or its prodromal phase.

## 4. Perspectives

Efforts can be directed toward the identification of lipid biomarkers, such as LPS and SCFA, both specific to the intestinal microbiota and which may be related to the development and progression of AD. These biomarkers have potential in early diagnosis and disease monitoring. Additionally, clinical studies must be conducted to investigate dietary interventions that aim to modify the composition of the gut microbiota in individuals at risk of developing AD, or those already diagnosed with the disease. This includes exploring diets, such as the MD and plant-based diet, which promote the maintenance of a healthy microbiota profile.

## 5. Conclusions

The profile of dietary lipids consumed has been proposed as a potential risk factor in the pathogenesis of AD and related dementias, but the evidence remains uncertain. Fatty acids have important effects on brain inflammation, synaptic plasticity, and memory. Lipids play a variety of essential roles in the healthy functioning of the brain. However, it is important to choose lipid sources like omega-3 fatty acids, while limiting the consumption of saturated and trans fats, which are associated with adverse effects on brain and overall health.

It is important to highlight that the composition of the GM is influenced by diet. Therefore, a healthy dietary pattern is an efficient way to prevent or delay the progression of AD. The MD, being rich in monounsaturated and polyunsaturated fats, could act preventively or provide relief from symptoms by reversing neuroinflammation and modifying the intestinal and blood–brain barriers in AD. On the other hand, the Western diet, characterized by high consumption of saturated and trans fats, combined with a sedentary lifestyle, inadequate sleep, and chronic stress, could intensify the progression of AD by promoting dysbiosis and alterations in the intestinal and blood–brain barriers, as well as chronic inflammation.

## Data Availability

Not applicable.
